# Editorial: Adaptive coping strategies for rehabilitation of people with non-specific chronic lower back pain or non-specific chronic neck pain

**DOI:** 10.3389/fresc.2025.1551777

**Published:** 2025-03-14

**Authors:** Chinonso Nwamaka Igwesi-Chidobe, Loveness Anila Nkhata

**Affiliations:** ^1^ Faculty of Health Studies, School of Allied Health Professions and Midwifery, University of Bradford, Bradford, United Kingdom; ^2^ Global Population Health (GPH) Research Group, University of Nigeria, Nsukka, Nigeria; ^3^ Division of Physiotherapy, Department of Health and Rehabilitation Sciences, Faculty of Medicine and Health Sciences, Stellenbosch University, Cape Town, South Africa; ^4^ Department of Physiotherapy, School of Health Sciences, University of Zambia, Lusaka, Zambia

**Keywords:** adaptive, coping, chronic low back pain, chronic neck pain, disability, pain, quality of life

**Editorial on the Research Topic**
Adaptive coping strategies for rehabilitation of people with non-specific chronic lower back pain or non-specific chronic neck pain

## Background

Pain coping—the use of strategies to manage the external or internal demands placed on a person by pain to reduce its impact on resources—mediates the impact of pain on disability and quality of life (1). There is uncertainty about which coping strategies improve clinical outcomes for people with non-specific chronic lower back pain (CLBP) or neck pain because coping strategies appear to be maladaptive or adaptive in different contexts. For instance, praying and hoping can reduce pain intensity (2) but can also increase anxiety and reduce range of movement (3). Similarly, coping self-statements can improve range of motion (3) or have no clinical impact (2). Coping strategies are believed by some authors to be unimportant after controlling for catastrophizing and pain self-efficacy (4). This may explain the limited research on coping strategies for back and neck pain in recent years. A contributory factor to the uncertainty about the importance of coping strategies could be because different coping strategies may have different impacts in different contexts and for different individuals. Moreover, outcome measures of current coping strategies may have limited applicability in non-western cultures (5).

This research topic aimed to bring together experts in chronic pain coping to investigate useful coping strategies that improve pain, disability, and quality-of-life in adults with CLBP or non-specific chronic neck pain. We invited systematic reviews, randomized controlled trials (RCTs), quasi-experimental studies, cohort studies, case control studies, cross-sectional studies, and qualitative studies. Findings were intended to inform the rehabilitation of people with non-specific chronic CLBP or neck pain.

## Findings from the research topic papers

Four studies [three cross-sectional studies and one piece of secondary qualitative research], all focused on back pain, contributed to this research topic.

The study by Fehrmann et al. investigated styles of coping with pain. This included three maladaptive coping styles: fear-avoidance, distress-endurance, and eustress-endurance. It also included one adaptive coping style: adaptive responders. Only 15% of the 137 Austrian patients with chronic back pain in the study used the adaptive coping style. The adaptive responders demonstrated more coping resources and had the lowest risk of pain chronification. The coping resources included exercising/sports (e.g., “exercising regularly is good for me”), hobbies/interests (e.g., “taking time to read a book feels good”), coping with stress/setting boundaries (e.g., “I am always able to delegate work”), positive attitudes (e.g., “complaining would only enhance the suffering”), social environment (e.g., “I’ve got a wonderful support system”), hope/motivation (e.g., “it will get better”), and external explanatory models (e.g., “both my parents also suffered from back pain”). They also perceived less job stress and reported fewer workplace problems, which could be linked to a better social support system at work. However, causality cannot be established by the cross-sectional design of the study.

Liu et al. found that Chinese nurses with a history of low back pain who had attended a prevention training program had a higher ability to prevent recurrence of occupational low back pain and chronicity of symptoms than those who had not. Self-efficacy was a protective factor that reduced coping barrier perceptions. This helped to overcome the intention-behavior gap, allowing the adoption of regular exercises to prevent chronic back pain. However, the perceived efficacy of the prevention training program was obliterated by long working hours that limited the time available for prevention behaviors such as rest and exercise. However, outcomes were based on the nurses’ perception of prevention rather than actual measured prevention of symptoms and chronicity. Moreover, the ergonomics education focus of the training program may not actually prevent back pain (6–9) and causality is constrained by the cross-sectional design of the study.

The study by Terfe et al. found that shorter working hours, regular physical activity, understanding the causes of low back pain, and less perceived job stress and job dissatisfaction reduced the occurrence of back pain among Ethiopian traditional cloth weavers. However, it is unknown whether other coping strategies were adopted that could have explained these outcomes. Moreover, causality is prohibited by the cross-sectional design of the study.

Igwesi-Chidobe et al. explored adaptive and maladaptive coping strategies used by Nigerian and Zambian adults with CLBP. Adaptive coping strategies included cognitive appraisal of CLBP as non-threatening, ongoing perceived ability to manage CLBP, adopting physical (including exercise) and psychosocial (including social support) coping strategies perceived as effective, and an expectation of a future improvement in coping outcome. The study found that most people used a mix of adaptive and maladaptive coping strategies that appeared to be driven by physical, psychological, or social pressures and the perceived success or failure of the coping strategy currently in use at particular moments. Another finding is that some coping strategies had both positive and negative aspects e.g., spirituality as a way of promoting inner peace vs. expectation of spiritual healing that did not happen or short-term use of pain medication for symptomatic relief vs. dependence on pain medications for daily functioning. Some coping strategies that were effective for some participants did not work for others, e.g., manual and cold therapies. Avoiding or reducing sustained manual work appeared useful for participants whose manual work aggravated their back pain. However, the usefulness of coping strategies were based on individuals’ perceptions rather than objectively measured constructs.

## Remaining gaps and a call for action

No studies on neck pain were submitted for this research topic. Consistent across these studies is the usefulness of regular exercise/physical activity, social support at work and in personal life, managing workload and stress, and positive cognitions and expectations about back pain. Self-efficacy may promote adaptive coping but sufficient time for recouping coping resources may be needed for self-efficacy to work. The pattern of utilization of coping strategies and their causal associations with clinical outcomes remain unknown. For example, it remains to be determined whether there will be different clinical outcomes associated with the simultaneous use of maladaptive coping strategies and adaptive coping strategies compared with the use of only adaptive coping strategies. Prospective studies and RCTs that utilize outcome assessments that capture patterns of utilization of coping strategies to determine their impact on pain, disability, and quality of life are required. Rehabilitation programs promoting the use of individual-specific adaptive coping strategies while discouraging the use of individual-specific maladaptive coping strategies using a case-by-case analysis may be needed to clarify the mechanisms of action and clinical impact.
